# Cytopathic effects in Mimivirus infection: understanding the kinetics of virus-cell interaction

**DOI:** 10.1590/0074-02760230186

**Published:** 2024-07-22

**Authors:** Gabriel Henrique Pereira Nunes, Juliana dos Santos Oliveira, Victor Alejandro Essus, Allan J Guimarães, Bruno Pontes, Juliana Reis Cortines

**Affiliations:** 1 Universidade Federal do Rio de Janeiro, Instituto de Microbiologia Paulo de Góes, Departamento de Virologia, Laboratório de Virologia e Espectrometria de Massas, Rio de Janeiro, RJ, Brasil; 2 Universidade Federal Fluminense, Instituto Biomédico, Departamento de Microbiologia e Parasitologia, Niterói, RJ, Brasil; 3 Universidade Federal do Rio de Janeiro, Instituto de Ciências Biomédicas & Centro Nacional de Biologia Estrutural e Bioimagem, Rio de Janeiro, RJ, Brasil; 4 University of Connecticut, Department of Chemistry, Storrs, CT, USA

**Keywords:** giant virus, virus and host interaction, cytopathic effect, Mimivirus, Tupanvirus, replication cycle

## Abstract

**BACKGROUND:**

Giant viruses have brought new insights into different aspects of virus-cell interactions. The resulting cytopathic effects from these interactions are one of the main aspects of infection assessment in a laboratory routine, mainly reflecting on the morphological features of an infected cell.

**OBJECTIVES:**

In this work, we follow the entire kinetics of the cytopathic effect in cells infected by viruses of the *Mimiviridae* family, spatiotemporally quantifying typical features such as cell roundness, loss of motility, decrease in cell area and cell lysis.

**METHODS:**

Infections by *Acanthamoeba polyphaga mimivirus* (APMV), Tupanvirus (TPV) and M4 were carried out at multiplicity of infection (MOI) 1 and MOI 10 in *Acanthamoeba castellanii*. Monitoring of infections was carried out using time lapse microscopy for up to 72 hours. The images were analyzed using ImageJ software.

**FINDINGS:**

The data obtained indicate that APMV is the slowest virus in inducing the cytopathic effects of rounding, decrease in cell area, mobility and cell lysis. However, it is the only virus whose MOI increase accelerates the lysis process of infected cells. In turn, TPV and M4 rapidly induce morphological and behavioral changes.

**MAIN CONCLUSIONS:**

Our results indicate that mimiviruses induce different temporal responses within the host cell and that it is possible to use these kinetic data to facilitate the understanding of infection by these viruses.

Viruses are classified as obligate intracellular pathogens due to the lack of their own machinery for energy production, synthesis of genetic material (RNA and DNA) and protein, thus being dependent on the transcription and translation molecular apparatus of cellular hosts for their replicative cycle to occur.^1^ Such dependency makes viruses masters in hijacking the cellular machinery for their own benefit, often negatively affecting the host. Interference with the complex signaling pathways that govern cellular homeostasis can lead to visible morphological changes in cells during virus replication cycle, known as cytopathic effect (CPE). The formation of CPEs depends on distinct parameters, such as host cell lineage, viral species, culture conditions, multiplicity of infection (MOI) and time, among others.^2^ As such, it can offer important insights into virus biology, virus-cell interaction mechanisms, and be used to track the progress of infections in the laboratory. Furthermore, CPEs are routinely used as a gold standard method after the isolation of new viral species, as they can function as indicators for quantification and monitoring these infections. Thus, it can be an important aspect in the identification of new viruses, including giant viruses (GVs).^3^ ^,^ ^4^ ^,^ ^5^ ^,^ ^6^ This is even more relevant when the CPEs observed are particularly unique in their occurrence, as is the case with cellular morphological changes associated with some GVs.^1^ ^,^ ^7^

GVs are the most recent members added to the virosphere with the first specimen, the *Acanthamoeba polyphaga mimivirus* (APMV), being discovered in 2003.^8^ Diverging from the size standards previously established for viruses, APMV displays a pseudoicosahedral viral particle between 450-500 nm.^8^ ^,^ ^9^ These viruses are mostly classified as amoeba parasites, being internalized by phagocytosis. Currently, several GVs have been described after the discovery of APMV, as viruses of icosahedral (*e.g.*, *Mimiviridae*, *Marseilleviridae*, *Faustoviridae*, *Pacmanvirus*, *Medusaviridae*), oval (*e.g.*, *Pandoravidae*, *Pithoviridae*, Cedratvirus, Orpheovirus) or spherical (Mollivirus) morphologies.^10^ ^-^ ^17^ Overall, GV replication follows precise steps, including virus adhesion, entry into the cell by phagocytosis and localization within the phagosome, with the subsequent delivery of the genome to the cytoplasm, formation of the viral factory, assembly of viral particles, and the release of new particles.^10^ ^,^ ^18^ The CPEs associated with infection by Mimivirus, Marseillevirus, Pandoravirus, Pithovirus, Cedratvirus, Pacmanvirus, and Medusavirus in *Acanthamoeba* spp. or by Faustovirus in *Vermamoeba vermiformis*, consist mainly of loss of adhesion/decrease in cell motility, cell rounding, and lysis at the end of the process. On the other hand, it is important to highlight the differences between each virus in the induction of CPEs, either by the kinetics of how the process occurs throughout the infection or the relative frequency of the process.^19^ ^,^ ^20^ These pieces of information help to delimit important stages of infection by GVs, through the temporal characterization of CPEs and, from this, can be used as a parameter for new studies of virus-cell interaction.

In this scenario, where there is little detailed information, the main factors of individual characterization of the cycles would be the periods of onset of cell morphology alteration and cell lysis. For the first parameter, there is a description of rounded cells after 30 min of infection for Marseilleviruses^21^ ^,^ ^22^ and after 8 h of infection for Pandoraviruses.^23^ Molliviruses represent a peculiar case, as they are the only GVs whose infection does not promote changes in cell morphology normally associated with GVs’ replication/infection during the infective process.^24^ ^,^ ^25^ However, in the infection generated by Medusavirus, cells were identified as both rounded or unrounded upon 48 h.^26^ For the second parameter, in the case of Marseillevirus, Pandoravirus, Pithovirus and Cedratvirus, cell lysis starts within 10 h of infection.^14^ ^,^ ^16^ ^,^ ^18^ ^,^ ^27^ ^,^ ^28^ However, the process of releasing newly assembled particles in the infection of Pacmanvirus and Faustovirus exhibits very specific times, being 6 and 18 h respectively.^11^ ^,^ ^18^ ^,^ ^29^ There are also viruses that do not induce cell lysis, as is the case with Molliviruses, which exit the cells via exocytosis.^18^ ^,^ ^24^ ^,^ ^30^

Structural and genomic features are widely used as identification mechanisms for virus species.^1^ ^,^ ^7^ However, we can highlight the importance of infection kinetics data and the evaluation of CPEs to determine viral infections. Thus, research on the intricacies of changes induced by GV infection may shed light on important stages of its cycle and on molecular aspects of virus-cell interaction that have not yet been described. To understand the formation of CPEs in viral infections it is necessary to evaluate the same parameters in healthy cells. Trophozoites of the amoeba *Acanthamoeba castellanii* display the ability to move using membrane protrusions called pseudopods, and cell sizes vary between 15 and 30 micrometers. In culture flasks, they grow adhered to the surface and move intensely. These parameters are acutely altered during infections by GVs, as a direct result of the CPEs. The goal of this study was to evaluate the kinetics of infection from the formation of CPEs, spatiotemporally analyzing the following parameters of cellular alteration: loss of typical cellular morphology (or rounding), alteration in the locomotion capacity, loss of area and, finally, cell lysis. Our analyses were carried out from infections by 3 viruses of the *Mimiviridae* family: Tupanvirus (TPV), APMV and the APMV-derived (M4), in *A. castellanii* cells. APMV was the first giant virus, discovered in 2003.^8^ TPV is a giant virus found in Brazil and has a cylindrical tail next to its capsid, in addition to having the most complete translation apparatus among known viruses.^4^ M4 is an APMV mutant that originated from consecutive passages in the laboratory, which culminated in an accumulation of mutations generating viruses without fibrils or in extremely reduced quantities.^31^ The occurrence of CPEs and the consequent structural changes to cells were observed mainly through time-lapse microscopy analysis during the period between the onset of infection and cell lysis. Therefore, we highlight the differences between infections and standardize the kinetics data for each species, with the goal of facilitating the evaluation of infections in laboratory routines.

## MATERIALS AND METHODS

*Cell culture -* Cells of *A. castellanii* (ATCC 30011) were cultured at 28ºC in 75 cm^2^ cell-culture flasks containing Peptone, Yeast Extract, and Glucose medium (PYG, ATCC Medium 712). Cells were passaged every 48 h to achieve highest viability.

*Giant virus purification* - *A. castellanii* cells were cultured as a confluent monolayer (5 x 10^6^ cells/mL). TPV, APMV or M4 were added at a MOI of 1:1. Cultures were kept at 28ºC. After 72-96 h, cultures were collected and centrifuged at 3500 xg for 10 min to remove cellular debris. The supernatant was collected for filtration through a 1.2 µm membrane to retain cellular debris. The filtered was carefully placed in a 22% sucrose cushion for centrifugation at 36,000 ×g for 30 min. The obtained virus pellet was resuspended in sterile phosphate-buffered saline (PBS).

*Time-lapse microscopy* - For time-lapse microscopy acquisitions, 3 x 10^5^ *A. castellanii* cells were seeded in a 35 mm culture dish and left at 28ºC for at least 2 h for adhesion. Next, the culture medium was removed and 2 mL of a new medium was added. The GVs were added at MOI 1 or MOI 10. After each GV addition, the culture dish was transferred to a culture chamber adapted to an inverted Leica DMi1 microscope (Leica) under controlled temperature (28ºC). For 72 h, phase-contrast images of the same field were captured every minute using a FLEXACAM C1 CCD camera (Leica). The images of each experimental condition were integrated into videos and analyzed using ImageJ software (National Institute of Health, Bethesda, MD, USA). Further details on each experiment will be discussed below.

*Cell rounding analysis* - For each experimental condition, the amoeba cell rounding over time was quantified. For such, as one of the cells became rounded, it was marked using the ‘’Multi-point’’ tool in ImageJ. The following criteria were used to identify rounded cells: absence of locomotion, absence of cell projections and round shape. For each marked cell, the criteria were recorded. Then, a plot of the mean number of rounded cells (normalized by the total number of cells) over time, t (h) was obtained for each experimental condition (colored dots). *τ*_*R*_ was defined as the time required for 50% of the amoeba cells to become round among all the cells of that specific condition, and it was determined based on the best fit (colored curves) obtained according to the following equation:

$$Y\left(t\right)=a+(b-a)/[1+{\left(t/\tau_{R}\right)}^{c}]$$

(1), where *a* and *b* are respectively the top and bottom plateau values of the Y-axis and *c* is the slope factor. The errors in the plots (light colored region) represent the standard error of the mean (SEM). The curve fits were performed using GraphPad Prism software (GraphPad Software).

*Cell motility analysis* - For each experimental condition, amoeba cell motility over time was quantified. The analyses were performed by measuring the displacement of 15 amoebas during the first 15 min of a total time interval of 100 min. Thus, every 100 min, 15 cells were monitored for the first 15 min. Values were obtained until all cells were completely immobile (displacement = 0). Measurements were performed using ImageJ ‘’Straight’’ tool, set to ‘’segmented line’’ and ‘’line Width’’ value 3. The total displacement was obtained by marking the amoeba position in the first frame and linking it with the amoeba position in the next frame and so on until the desired 15 min was achieved. The average velocity for each time point was determined based on the average displacement of the 15 different amoeba cells over the 15 min interval. A plot of the average velocity (dots in the plots) over time, t (h) was obtained for each experimental condition. Finally, for each plot a curvefit was defined based on the following equation:

$$G\left(t\right)=\left(m-n\right)\times e\left(-kt\right)+n$$

(2), where *m* is the Y-value when *t* is zero, *n* is the Y-value at infinite times, *k* is the rate constant and τ_*v*_ is the time required for 50% loss of velocity and it is computed as *ln*(2)/*k* The errors in the plots (vertical bars at each time point) represent the SEM. The curve fits were performed using GraphPad Prism software.

*Cell area analysis -* For each experimental condition, the area of amoeba cells over time was quantified. Values were obtained by marking the area of 15 cells throughout the infection. Measurements were taken from 0 to 2000 min post-infection, at intervals of 100 min for each measurement. Measurements were performed using ImageJ ‘’Straight’’ tool, adjusted to ‘’segmented line’’ and ‘’line Width’’ value 3. The area was obtained by marking the contour of the cells.

The average area value for each time point was determined based on the areas of the 15 different amoebas. A plot of the average area (dots in the plots) over time, t (h) was obtained for each experimental condition. Finally, for each plot a curvefit was defined based on the following:

$$F\left(t\right)=\left(p-q\right)\times e\left(-rt\right)+q$$

(3), where *p* is the Y-value when *t* is zero, *q* is the Y-value at infinite times, *r* is the rate constant and *τ*_*A*_ is the time required for 50% loss of area and it is computed as *ln*(2)/*r*. The errors in the plots (vertical bars at each time point) represent the SEM. The curve fits were performed using Prism software.

*Cell lysis analysis* - For each experimental condition the amoeba cell lysis over time was quantified. The analyses were done using the ImageJ software. Lysed cells were marked using the ‘’Multi-point’’ tool in ImageJ. Lysed cells were identified by loss of rounded shape and release of internal content. Then, a plot of the mean number of lysed cells (normalized by the total number of cells) over time, t (h) was obtained for each experimental condition (colored dots). *τ*_*L*_ was defined as the time required for 50% of the amoeba cells to become lysed among all the cells of that specific condition, and it was determined based on the best fit (colored curves) obtained according to the following equation:

$$W\left(t\right)=a+(ba)/[1+{(t/\tau }_{L\;}{)}^{c}]$$

(4), where *a* and *b* are respectively the top and bottom plateau values of the Y-axis and *c* is the slope factor. The errors in the plots (light colored regions) represent the SEM. The curve fits were performed using GraphPad Prism software.

## RESULTS

**Fig. 1:**
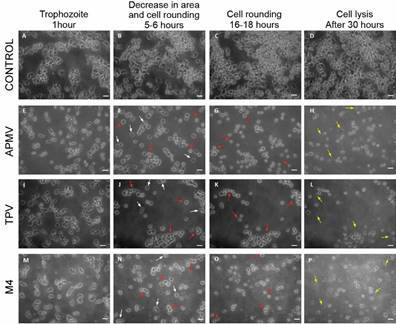
*Acanthamoeba castellanii* at different stages of giant virus infection showing the steps of cytopathic effects. Images of *Acanthamoeba polyphaga mimivirus*, Tupanvirus and M4, induced infections at multiplicity of infection 10 were selected. A-D: uninfected *A. castellanii* trophozoites were used as control. Cell morphology did not change over time of infection. E, I and M: early stages of infection where cells were still in trophozoite morphology. F, J and N: initial stage of the cytopathic effects, where there was a mixture of fully rounded cells (red arrows), with trophozoite cells and also cells between these two stages (white arrows). G, K and O: consolidation of the cell rounding in all cells in the field (red arrows). H, L and P: beginning of cell lysis. Yellow arrows indicate cell debris from lysed cells, red rows indicate fully rounded cells (before cell lysis). The scale bar represents 40 µm.

*TPV, APMV and M4 infections induce the same CPEs: rounding, loss of mobility, area loss and cell lysis in A. castellanii cells* - Most GV infections (including Mimivirus) induce the same CPEs, which are defined by the morphological changes in amoeba trophozoites, which culminate in cell rounding.^4^ ^,^ ^18^ ^,^ ^32^ However, a detailed kinetic characterization of the process has yet to be established. Therefore, time-lapse microscopy images were taken throughout cycles of mimiviruses infections. Analysis of infected cells showed that CPE occurrence/formation is a continuous process and that it can take a few minutes from the first signs of cellular stress to full rounding, resulting in cell lysis at the end of the virus replication cycle. Using the obtained data and analyzing the results, we delimited the steps that will be addressed throughout this study: the gradual loss of cellular motility culminating in total stagnation (1); loss of cell area throughout the infection (2); rounding of infected cells (3) and finally cell lysis (4). Supplementary data (Video 1) represents a typical infection process, clearly showing the four steps that will be quantified throughout this work. In contrast, a representative control condition is presented in Supplementary data (Video 2). Some morphological features were comparatively highlighted. The control condition did not show evident morphological changes, except for an already expected increase in number of cells over time (Fig. 1A-D). The infected condition (Fig. 1E-P) clearly presented decreases in cell areas, cell rounding (red arrows) and cell lysis (yellow arrows). White arrows indicate cells still in the trophozoite stage along the rounding process [Fig. 1, Supplementary data (videos 1 and 2)].

In the following sections, we will present more robust spatiotemporal quantifications of these mentioned CPE steps, comparing infections for the three different GVs used in this study: TPV, APMV and M4.

**Fig. 2:**
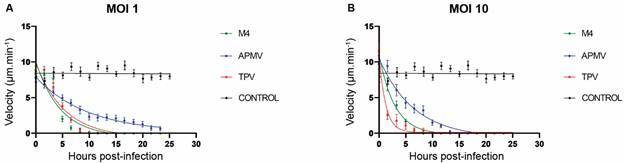
cell motility throughout *Acanthamoeba castellanii* infections by giant virus. Graphs showing the variation in velocity of *A. castellanii* cells infected by *Acanthamoeba polyphaga mimivirus* (blue), Tupanvirus (red) and M4 (green) at multiplicity of infection 1 (A) and 10 (B) over time. Uninfected *A. castellanii* cells were used as control (black curves in A and B). The dots represent the mean velocity value (with its standard error of the mean value represented by the vertical bar) for each timepoint and condition in the graph, while the curves represent the exponential fits according to Eq. (2).

*Analysis of motility loss kinetics* - Loss of cell motility is directly associated with the morphological changes that occur during GV-induced infections. As infection progresses, these amoeba cells lose the ability to generate projections and a decrease in locomotion velocity should be noted until they come to a complete halt. Thus, we next quantified the changes in velocity of amoebas infected with each of the three viruses used in this study, for MOI 1 and MOI 10. Two main parameters were obtained: the time to induce 50% loss of velocity and the time to induce total stagnation of all cells (Table and Fig. 2).

**TABLE:** Summary of the kinetic parameters obtained at each cytopathic effect (CPE) step of this study. Comparisons between the three viruses and the two multiplicity of infections (MOIs) evaluated

Virus	MOI	Time for 50% loss of velocity (h)	Time for total stagnation (h)	Time for 50% decrease in cell area	Time for 50% cell rounding (h)	Time for 100% cell rounding (h)	Time for 50% cell lysis	Time for 100% cell lysis
APMV	1	6.75 ± 2.80	24	5.50 ± 0.73	12.35 ± 0.10	24	37.22 ± 0.10	72
	10	4.54 ± 0.83	15	5.60 ± 0.85	9.16 ± 0.10	18	29.96 ± 0.03	44
TPV	1	3.70 ± 0.38	13	8.80 ± 1.50	9.14 ± 0.07	20	34.35 ± 0.03	43
	10	0.84 ± 0.23	10	3.96 ± 0.56	5.78 ± 0.04	16	34.50 ± 0.03	47
M4	1	3.16 ± 0.40	12	4.51 ± 0.59	8.76 ± 0.03	14	33.10 ± 0.20	64
	10	2.03 ± 0.23	8	4.40 ± 0.46	8.07 ± 0.04	16	40.60 ± 0.07	72

APMV: Acanthamoeba polyphaga mimivirus; TPV: Tupanvirus.

At MOI 1, APMV infections took the longest to induce 50% loss of velocity and total stagnation of cells, with 6.75 ± 2.80 h and about 24 h, respectively (Fig. 2A and Table). In contrast, TPV and M4 infections were much faster in promoting these effects, taking 3.70 ± 0.38 and 3.16 ± 0.40 h respectively to induce 50% loss of velocity and about 13 and 12 h respectively to induce total stagnation of cells (Fig. 2A and Table).

At MOI 10, APMV infections continued to be the longest to induce both the 50% loss of velocity and total stagnation, whose times decreased to 4.54 ± 0.83 and 15 h, respectively (Fig. 2B and Table). At the same MOI 10, TPV and M4 also showed reduction in the time required to induce 50% loss of velocity, showing respectively 0.84 ± 0.23 and 2.03 ± 0.23 h and about 10 and 8 h, respectively, for total cell stagnation (Fig. 2B and Table).

Overall, the observed kinetic values indicate that at MOI 1 APMV infections take the longest to induce 50% and total loss of velocity. TPV and M4 are fastest in inducing these effects and have similar times. In MOI 10 there is an acceleration in the induction times of 50% and a total loss of velocity. Furthermore, they clearly show the influence of increased MOI on the induction of CPE upon mimiviruses infections.

*Decrease in the overall cell area* - Another morphological change observed during the CPE is the reduction in the area of infected cells. Seeking to better understand this feature and to quantitatively follow the effects in size of *A. castellanii* cells throughout infection, we used our time-lapse microscopy videos to perform such analysis to establish the importance of the virus type used in this study and the MOI variations (1 or 10), together with an uninfected cells control.

The analysis of cell area kinetics was also based on two parameters. The first was to evaluate the difference between the highest and lowest mean area values in each experimental condition, which allowed us to calculate the percentage of area loss at the end of the infection. The second parameter was to identify the time point to induce a 50% reduction in the total area of cells. The obtained data were compared with each other and between the MOIs used.

**Fig. 3:**
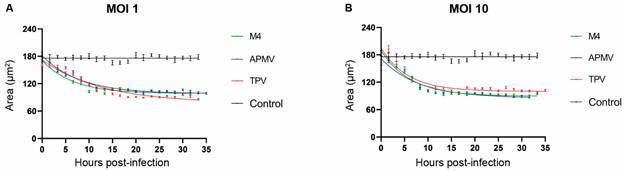
loss of cellular area throughout the infection of *Acanthamoeba castellanii* by giant virus. Graphs showing the variation in area of cells infected by *Acanthamoeba polyphaga mimivirus* (blue), Tupanvirus (red) and M4 (green) in infections with multiplicity of infection 1 (A) and 10 (B) over time. Uninfected *A. castellanii* cells were used as control (black curves in A and B). The dots represent the mean area value (with its standard error of the mean value represented by the vertical bar) for each time point and condition in the graphs, while the curves represent the exponential fits according to Eq. (3).

Regarding the difference in cell area, at MOI 1, APMV and TPV presented similar values, with a slight tendency for TPV to induce a larger difference. While APMV-infected cells lost about 45% (Fig. 3A, blue) of the total area at the end of the infection, TPV-infected cells lost about 47% (Fig 3A, red) and M4-infected cells lost about 39% (Fig. 3A, green). However, analysis of the times to induce 50% decreases in total cell areas showed APMV with 5.50 ± 0.73 h, TPV with 8.80 ± 1.50 h and M4 with 3.96 ± 0.56 h (Fig. 3A and Table). The uninfected control condition showed no area variation over time (Fig. 3A, black).

The same analyses were carried out for MOI 10. Comparing the loss of area, TPV- and APMV-infected amoebas both showed decreases of 42% in area, while M4 showed a decrease of 48% (Fig. 3B). The main differences, however, were the times for 50% decrease in total area. TPV accelerated the cell area reduction process to 3.96 ± 0.56 h, but the times were comparable for APMV and M4 infections, 5.60 ± 0.85 and 4.40 ± 0.46 h respectively (when compared to their respective times at MOI 1) (Fig. 3B and Table).

APMV and TPV infections have different responses to MOI increase in relation to area loss over time. Interestingly, both infections induce similar variations in cellular areas, as demonstrated by the percentage of area loss at the end of the process.

**Fig. 4:**
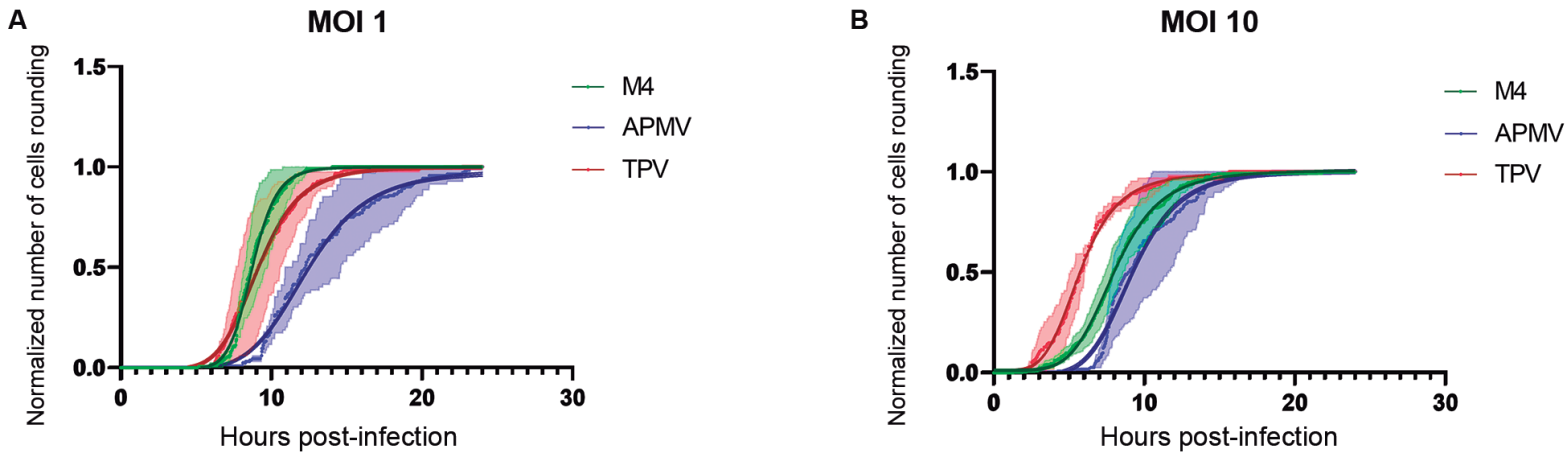
characterization of the rounding kinetics of *Acanthamoeba castellanii* cells infected by each of the three viruses used in this study. *Acanthamoeba polyphaga mimivirus* (blue), Tupanvirus (red) and M4 (green) at multiplicity of infection 1 (A) and 10 (B). The green, blue and red curves represent the normalized number of rounded cells (with its range of standard error of the mean values represented by the light green, red or blue region) over time. The dark green, dark blue and dark red curves represent the four-parameter logistic sigmoidal fits according to Eq. (1).

*Rounding kinetics show differences in the formation of classic mimiviruses CPEs* - While the overall steps during infection by mimiviruses seem apparently similar, there are striking differences in the time necessary for each step to be reached. At MOI 1, TPV and M4 were the fastest in inducing amoeba cell rounding, as indicated in the graphs of Fig. 4. For 50% amoeba cell rounding, TPV- and M4-induced infections took 9.14 ± 0.07 and 8.76 ± 0.03 h respectively, whereas APMV took 12.35 ± 0.10 h (Fig. 4A and Table). However, for 100% amoeba cell rounding, M4 infections were the fastest, reaching it at 14 h, while these time points for TPV and APMV were 20 and 24 h, respectively (Fig. 4A and Table).

The same parameters (cell rounding percentages) were also analyzed at MOI 10. The time required for 50% amoeba cell rounding during TPV and APMV infections were respectively 5.78 ± 0.03 and 9.16 ± 0.10 h (a decrease of approximately 3 h when compared with their respective values in MOI 1), whereas for the M4 infection was a decrease of less than 1 h when compared to the value obtained at MOI 1, with a time of 8.07 ± 0.04 h (Fig. 4B and Table). For 100% amoeba cell rounding at MOI 10, the time shortening patterns were also observed for TPV and APMV, registering the time points of 16 and 18 h respectively, conversely for M4, the time point was 16 h, an increase of about 2 h when compared to MOI 1 (Fig. 4B and Table).

The observed patterns are virus-independent and may indicate the relationship between the number of particles (even non-viable virions) and the development of cellular morphology changes over time.

*The kinetics of CPEs delimit the kinetics of cell lysis* - The end of the cell cycle is marked by the release of viral particles into the extracellular environment. In the case of mimiviruses, this occurs through cell lysis. Using time-lapse microscopy, we also quantitatively determined the lysis kinetics and compared the effects of MOI increase in infections by APMV, TPV and M4.

**Fig. 5:**
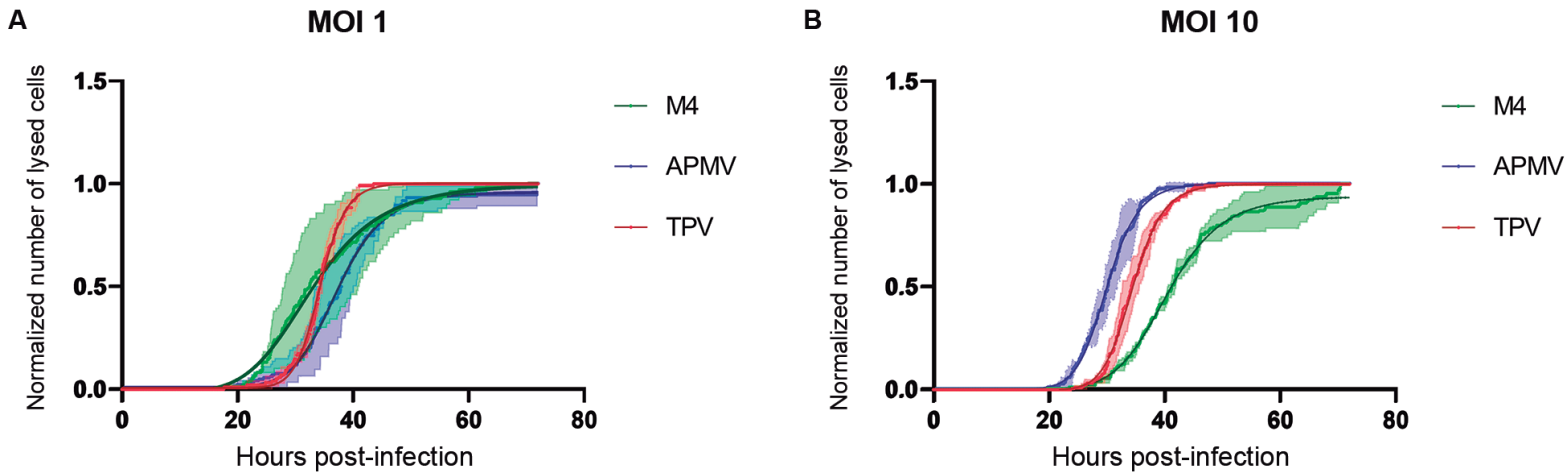
characterization of the lysis kinetics of *Acanthamoeba castellanii* cells infected by each of the three viruses used in this study. *Acanthamoeba polyphaga mimivirus* (blue), Tupanvirus (red) and M4 (green) at multiplicity of infection 1 (A) and 10 (B). The green, blue and red curves represent the normalized number of lysed cells (with its range of standard error of the mean values represented by the light green, blue or red region) over time. The dark green, dark blue and dark red curves represent the four parameter logistic sigmoidal fits according to Eq. (4).

Graphs of cell lysis over time showed APMV as the virus with the slowest process to induce amoeba lysis at MOI 1. The initial times for the onset of lysis were similar to those of TPV at MOI 1 but the kinetics were completely different (Fig. 5A): for both viruses, lysis started around 20 h after infection. While for M4 the process started 2 h earlier, around 18 h. The difference in lysis kinetics for each of the viruses was maintained throughout the entire infection process, *i.e.*, until all cells were lysed, indicating the end of the infection cycle. APMV was the virus with the longest replication cycle, lasting about 72 h at MOI 1 (Fig. 5A).

In APMV infections, increasing the MOI considerably decreased the total infection end time (lysis). The onset of lysis occurred at practically the same intervals (22 h). But at MOI 10 it took about 28 h less to complete the process (72 h at MOI 1 and 44 h at MOI 10). For 50% of lysed cells, the time also decreased in MOI 10, from 37.22 ± 0.10 (MOI 1) (Fig. 5A and Table) to 29.96 ± 0.03 h (MOI 10) (Fig. 5B and Table).

Increasing MOI for TPV and M4 delays the time for total cell lysis. TPV infections took about 4 h longer at MOI 10 for total lysis induction, going from 43 h at MOI 1 to 47 h at MOI 10 (Fig. 5). As for M4, the increase in time was about 8 h, going from 64 h at MOI 1 to 72 h at MOI 10) (Fig. 5A-B). The interval to initiate lysis and to reach lysis of 50% of the cells remained practically the same in infections of TPV MOI 1 and 10. In M4 infections, we observed an increase in the time to initiate lysis, going from 18 h at MOI 1 to 26 h at MOI 10. In addition, an increase in the time at which 50% of the cells were lysed was observed, changing from 33.10 ± 0.20 h at MOI 1 to 40.60 ± 0.07 h at MOI 10 (Fig. 5 and Table). For TPV infections 50% of cell lysis occurs in 34.35 ± 0.03 at MOI 1 and 34.50 ± 0.03 at MOI 10. This indicates that there really is a delay in the replication cycle with increasing MOI. As for the TPV, at first the delay occurs at the end of the infection since the onset of lysis occurs at the same time in both MOIs and the change in 50% of the lysed cells is almost null (Table). APMV proved to be the only virus to respond positively to the increase in MOI in relation to lysis times.

## DISCUSSION

The key infection times for some GVs have already been noted in the literature, as well as the CPEs formed by each infection.^12^ ^,^ ^19^ ^,^ ^20^ These data are mainly acquired by following the infection by microscopy or molecular analyses, such as gene expression data.^33^ ^,^ ^34^ ^,^ ^35^ However, the times obtained are based on punctual observations and absent from quantitative analyses, evaluating continuously throughout the process. Recently, some studies have investigated quantitative data on GV infections.^19^ ^,^ ^20^ These data, alongside the results shown in this work, help enrich our knowledge on the replicative cycle of GVs in amoebas, detailing the key infection times and the reflexes in the formation of CPEs in amoebas, through a continuous care approach, using smaller time intervals, which helps with detailed characterizations. Our quantifications also help to define patterns for each infection, favoring comparative analysis between different species, in addition to facilitating new studies on virus-cell interaction. In addition to the data already found in the literature for the TPV and, mainly for the APMV, which indicate the different stages of its replicative cycle and the important times for infections,^3^ ^,^ ^25^ ^,^ ^28^ here we show the first descriptive and quantitative data regarding the replicative cycle of the M4. This fibril-deficient mutant derived from APMV is often used as a control for APMV assays, particularly those involving the characterization of fibril functions.^31^ ^,^ ^32^ As fibrils are relevant in viral attachment, our hypotheses would be a longer cycle for M4, imagining an initial difficulty in the virus-cell interaction, as previously reported in the literature.^32^ ^,^ ^36^ However, our data based on CPE formation showed a completely different infection pattern between APMV and its mutant. M4 exhibited a faster replicative cycle and induced CPEs before APMV, independent of possible changes in viral attachment differences in fibrils, as previously reported by Boyer and colleagues.^31^ Based on the genetic differences already characterized between M4 and APMV, our data help indicate the possibility of different infection strategies. In addition to the mutations that affected the fibrils, as mentioned previously, M4 is a model that can be used as a base model for understanding how genetic changes arise according to the conditions in which the infection occurs over generations. Our data for M4 indicate an acceleration in the occurrence of cytopathic effects of rounding, loss of speed and loss of area, in addition to indicating changes in the final process of the cycle (cell lysis). This indicates how genetic variations in M4 can directly interfere with the infection times of these viruses. Based on the kinetic data presented here, the hypothesis was considered that the time variation may be related to the speed of assembly and production of new M4 particles, which could be accelerated by the absence of fibril production. In a way, this variation in replication could compensate for the possible difficulty in adhesion to cells due to the absence of fibrils, as had been previously thought. In turn, the toxicity of Mimivirus particles has already been determined by the presence of fibrils. Oliveira and collaborators demonstrated that fibrils trigger the TLR4 signaling pathway in lung cells, due to the composition of the fibrils and similarities with LPS.^37^ Thus, cells in the presence of fibrils could trigger toxicity responses, while M4, theoretically, would not trigger this signaling pathway. Another work that explored the emergence of mutated strains was done by Mueller and collaborators. Seeking to understand the formation of mutations in viral lineages they carried out a study with consecutive passages over a year with Lausannevirus in *A. castellanii*. The authors used allopatric, sympatric and competition conditions with a microorganism (*Estrella lausannensis*) to try to understand how mutations arise, their location in the genome and how this interferes with viral replication factors, such as total infection time and produced progeny after the end of the infection. Variations in the viral genome were recorded throughout the different populations created during the experiment period. Most mutations/deletions occurred in hypothetical proteins or with putative functions. Thus, it was observed that most conserved genes present in other giant viruses were not altered. As with M4, consecutive passages of Lausannevirus generated genetic alterations and differences in timing and viral production throughout the cycle. This approach helps to understand the evolution of giant viruses, the emergence of new variants, the role of conserved genes and the impact of mutant proteins in the replicative cycle. On the other hand, together with genomic data, we highlight the importance of quantifying the infection kinetics for the identification of recognizable infection patterns for each virus as it makes possible comparisons between mutant viruses.^38^ It is important to emphasize the need for new studies that seek to study molecular differences in APMV and M4 infection, to understand how mutations and deletions in the M4 genome interfere with each step of the replicative cycle. Boyer and collaborators described the genetic differences between APMV and its variants over successive passages until the formation of M4 (the last mutant to originate), whose genome showed a reduction of around 16% of the total, around 0.2 mb.^31^ The deleted or mutated genes were related to various biological functions, including carbohydrate metabolism, protein expression, DNA replication, recombination and repair, as well as metabolic and structural functions, viral morphogenesis and virus-cell interactions. In fact, to indicate the real influence of these mutations, molecular and functional investigations are necessary throughout the cycle.^9^ ^,^ ^31^ One of the new strategies is the use of genetic editing by CRISPR/Cas. This strategy was used to study evolution of Pandoravirus and thus to understand the ancestry relationships with other viruses and their hosts.^39^ Genome editing can be an interesting tool in the future to aid in the investigation of the function of the proteins altered by the mutations present in M4, showing how these are associated with the differences observed in its replicative cycle.

**Fig. 6:**
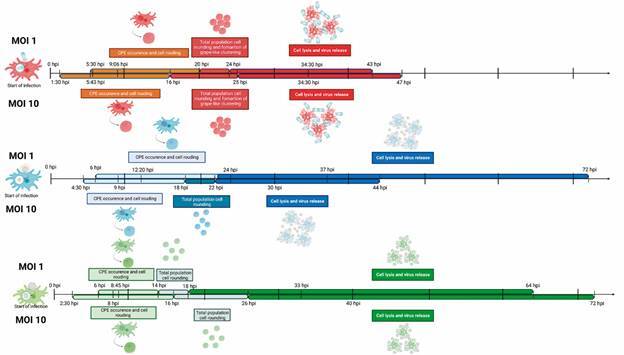
timeline characterization of cytopathic effects in Tupanvirus (A), *Acanthamoeba polyphaga mimivirus* (B) and M4 (C) infections in *Acanthamoeba castellanii* cells at multiplicity of infection 1 (top line) and 10 (bottom line). Description of the morphological changes of the cells throughout the infection and delimitation of the times of occurrence of each cytopathic effect (Table). hpi: hours post-infection.

When we associate the area loss data with rounding progression data, we adopt two different profiles. In those accompanied by TPV, the time interval for 50% of rounded cells corresponds to the same time interval in which cells arrive in the proportion of 50% of the total area lost. This kinetics may indicate a simultaneous process, where the loss of area is proportional to the advancement of the rounding process. On those compatible with APMV, the pattern seems to be different. The decrease in the area corresponding to 50% of the total area lost (half the difference between the start and end area) occurs before the 50% of the rounded cells. That is, this may indicate that area loss occurs mostly before the cells complete the rounding stage. These data indicate a difference in transition kinetics between trophozoite and round cell morphology, showing that this is a continuous and gradual process, making clear the difference in the induction of CPE in each infection (Fig. 6). Ben Yaakov et al.^19^ evaluated cytoskeletal alterations at key moments in the replicative cycle in APMV infections in *Acanthamoeba polyphaga*. APMV infections in *A. polyphaga* resulted in morphological changes in cells around 4 h post-infection, according to data obtained in flow cytometry. Between 4-6 h, fragmentation of microtubules was observed as well as retraction of actin filaments present in filopodia and pseudopodia.^19^ Alterations in the cytoskeleton are directly related to the rounding processes and, consequently, to the loss of cellular mobility.^19^ ^,^ ^20^ Our kinetic data agrees with those shown for the APMV, as at MOI 1 the cell rounding starts at about 6 h and at MOI 10 at about 4:30 h. In this interval, decreases in the cellular area and velocity were also observed, agreeing with the times indicated for changes in amoeba.

The complete rounding of the cells is directly related to the loss of area, as described above, but it is also the factor that leads to the loss of amoeba mobility. In APMV infections, the average cell rounding curve follows the key times (50% and 100% cells rounded) against the loss of displacement curve (50% velocity loss and zero displacement velocity). This pattern occurs in both MOIs, varying only the period in which they occur (Table). For TPV infections, cells first become immobile and only then complete the rounding process. The 50% times in each of the analyses maintain the same pattern, because the loss of 50% of displacement velocity occurs before the 50% rounded cells in MOI 1 and MOI 10. M4 follows the same pattern as TPV; cells first become immobile and only then complete the rounding process. It had already been reported that TPV infections at MOI 100 delay the rounding and formation of the unique Tupan-derived CPE, called grape bunches.^40^

The formation of CPEs is the visualization of virus-cell interactions and cellular mechanisms of response to infection. The induction of morphological and behavioral changes in amoeba may be associated with viral dispersion, facilitating the spread of the virus to new areas after the end of the replicative cycle.^20^ ^,^ ^32^ TPV-infected cells tend to round more quickly than APMV-infected ones. These results are consistent with those observed in previous studies, which show the formation of the CPE around 4-6 h for TPV and around 6-8 h for APMV.^33^ ^,^ ^35^ ^,^ ^40^ The ‘’delay’’ in the appearance of CPEs in APMV infections favors a greater displacement of these cells compared to those infected with TPV, which in turn favors a faster rounding and the formation of ‘’grape bunches’’.^40^ Therefore, APMV-infected cells can still propagate in the environment even when already infected, at least until the most advanced stages of infection. In contrast, TPV infections follow an opposite pattern. Our data show that, regardless of the MOI, TPV-infected cells tend to round more quickly than APMV-infected ones causing an early and sudden drop in cell motility, around 2 h after infection. For TPV infections with MOI 10, this occurs in less than 1 hour of infection. This may be associated with another viral dispersal strategy, starting with the formation of grape clusters. In TPV-infected cells, this exclusive CPE occurs due to the overexpression of viral and cellular mannose-binding proteins (MBPs).^40^ Oliveira and collaborators, 2019, argue that such phenomena can be associated with mechanisms of viral dispersion, where infected cells would adhere to non-infected cells. In this context, uninfected trophozoites would act as dispersion agents, dispersing infected cells.^40^ Moreover, it would also become a potential host after the release of new viral particles due to its proximity to the infected cell and the consequent exposure to the viral progeny. Furthermore, the interaction of these cellular aggregates with other types of cells in nature cannot be ruled out, as little is known about the relationship of giant viruses with other potential hosts, and their ability to adhere to different surfaces.^32^ ^,^ ^34^ ^,^ ^41^ It is important to point out though that among the mimiviruses studied so far, TPV is the one that has the widest range of hosts to date.^4^ ^,^ ^34^ Also, mimiviruses have a great ability to attach to other biological model.^32^ The same is true for TPV which can stick to other cell types and biological surfaces through the interaction of their fibrils.^4^ ^,^ ^32^ Other viruses form cell aggregates, as is the case with hokutovirus.^20^ ^,^ ^42^ The formation of cell rounding and cell aggregates can be explained in two different contexts: association of behavior change as a response to amoeba defense mechanisms and; response induced by viral infection as a mechanism to favor viral dispersion.^5^ ^,^ ^20^ ^,^ ^40^ From this, we highlight the need for new data to better characterize the viral strategies induced throughout the infection and their cause-and-effect relationship.

Viral infections are regulated by several factors that modulate virus-cell interactions. In the case of giant viruses, we can mention the structural and genomic complexity, ancestry and evolution, and possible host range, in a wide spectrum of still unknown parameters. Data on the kinetics of infection based on the formation of CPEs are presented as an excellent approach for new studies on the stages of infection.^19^ ^,^ ^20^ ^,^ ^43^ These analyses were carried out by evaluating cell by cell, continuously from the beginning to the end of the replication process (lysis), with measurements in short periods of time to characterize the replicative cycle quantitatively, culminating in kinetic data. Thus, this approach generated more accurate data, different from those available in the literature to date, as here we quantified CPE formation through a continuous minute-by-minute approach to the entire infection. Our initial analyses were carried out in the absence of any comparison with pre-established data, to assemble a pattern without any external interference to our experiments. This was done with the goal of indicating the divergences and possible experimental factors that interfere with and modulate the formation of CPEs, as well as identifying the similarities and divergences in relation to other strategies used to delimit infection parameters. In this scenario, Fukaya and collaborators, in 2023, showed the morphological changes that occur in the nucleus and vacuoles of *A. castellanii* infected by four different GVs, with a focus on Medusavirus. The authors showed an analysis that approaches the presence/absence and size of the nucleus in a quantitative way over the time of infection, comparing data between viruses and non-infected cells. With this, the authors could compare the formation of CPEs with morphological changes inside the cell. In this way, indicating the times of nucleus and cytoplasm rearrangements, with the formation of the viral factory, which are induced by infections with some GVs.^44^

Abrahão and collaborators showed that TPV induces the formation of CPEs in cells even in the absence of replication or entry of these viruses into cells, with data mainly using other amoeba genera.^4^ ^,^ ^34^ Oliveira and collaborators indicated that TPV and APMV induce activation of the TLR4 pathway in pulmonary epithelial cells, also in the absence of viral replication. The authors showed that TPV has a greater cytotoxicity than APMV, generating a greater response through the TLR4 pathway.^37^ Based on this knowledge, we hypothesize that TPV appears to be more ‘’toxic’’ after recognition/contact with cells. We hypothesize that this may explain why TPV induces cellular changes prior to APMV and M4, at similar MOIs and at early times of infection. In addition, we can discuss the possible relationship between non-infectious particles of Mimivirus in the induction of CPEs. In this case, it would be mainly reflecting the molecular responses triggered by virus-cell recognition, or by molecular mechanisms associated with steps prior to the expression of viral genes by the host. Schrad and collaborators in 2020 assessed, by mass spectrometry, different molecules present in Mimivirus particles such as APMV and Sambavirus. The authors found the presence of proteins and mRNAs, in addition to indicating the possible related biological functions.^45^ These data show the diversity of the content carried into the cells, so that such molecules, many without a defined function, can be associated with the modulation of responses even in the absence of replication, as already reported for some mimiviruses.^4^ ^,^ ^37^ ^,^ ^45^ We highlight the importance of further studies that seek to understand the composition of the particles of these GVs and the content that is carried into the interior of the host. It is known, for example, that mimiviruses have a range of proteins, RNAs and other molecules that are directed to the cytoplasm by the viral seed and that have different biological functions even in the absence of expression of viral proteins by the cell.^45^

Other factors may affect virus-cell interaction mechanisms, as is the case with virophages, a remarkable feature present in the world of GVs. Virophages are viruses that use infection by GVs to produce new viral particles. These “small” viruses are internalized together with some GVs.^46^ ^,^ ^47^ ^,^ ^48^ In the case of mimiviruses, they are believed to bind to fibrils.^36^ ^,^ ^38^ After internalization, they use the viral factory generated by the giant virus infection to synthesize their proteins and replicate their genetic material. Virophage-associated Mimivirus infections have been reported to have affected infection fitness, decreasing the number of new particles formed and delaying some infection processes.^47^ ^,^ ^49^ ^,^ ^50^ The presence of virophages in GV preparations was not followed during GV experiments performed here. Therefore, just as we highlighted the importance of our kinetics data to understand aspects of cell virus interaction, we can also highlight the importance in understanding the mechanisms between GVs, host cells and virophages and how these entities affect the replicative cycle. Our kinetics data favor the identification of quantitative patterns from CPEs analysis that can help to elucidate these different aspects of cell-virus interaction and identification of distinct CPEs (Fig. 6). Such as the presence of virophages, host cells and MOI, in addition to promoting a comparative analysis between some viruses.

In conclusion, our study showed the kinetics in the formation of CPEs in *A. castellanii* infected with three different mimiviruses: TPV, APMV and M4. The data obtained help to elucidate the infection through the morphological and behavioral alterations of the cells, as a result of the different molecular phenomena of virus-cell interaction. For this, the effects of rounding, loss of motility, loss of cell area and lysis, which characterize crucial stages of the replicative cycle from a visual perspective, were addressed. In addition, our comparisons with infections at different MOIs delimited the influence of adding more viral particles on the kinetics of CPE formation, showing the differences in responses induced by each virus. Quantitative analyses of CPEs can be an excellent strategy to be used in differentiating viral infections, in addition to being a starting point for molecular analyses. We emphasize here the need for further studies to elucidate the cellular signaling induced by infections that determine the formation of each of the CPEs evaluated here. We showed the variability of phenomena and cellular responses that can be obtained with similar approaches, from the addition of new variables such as new species, culture conditions, host cell and MOIs. Understanding the morphological variations generated by infections helps to define parameters for observation of the replicative cycle in laboratory routines. Our data, in addition to promoting comparative approaches, favor the standardization of CPE variations in with the characterization of GVs.
