# Supplementary material for: Cytopathic effects in Mimivirus infection: understanding the kinetics of virus-cell interaction
Source: Mem Inst Oswaldo Cruz. 2024 Jul 22;119:e230186. doi: 10.1590/0074-02760230186 (PMC11285858; doi:10.1590/0074-02760230186)
Supplement: Supplementary file 1 [file 1678-8060-mioc-119-e230186-s.pdf]

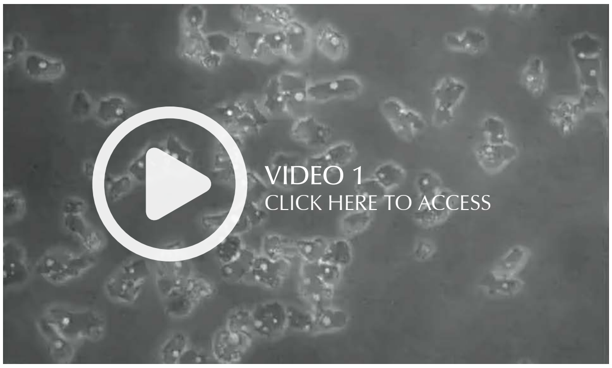

Video 1: Tupan virus infection in *Acanthamoeba castellanii* in multiplicity of infection 10 as an example of the images obtained for the analysis of infection kinetics by evaluating cytopathic effects (rounding, loss of area, loss of velocity and lysis).

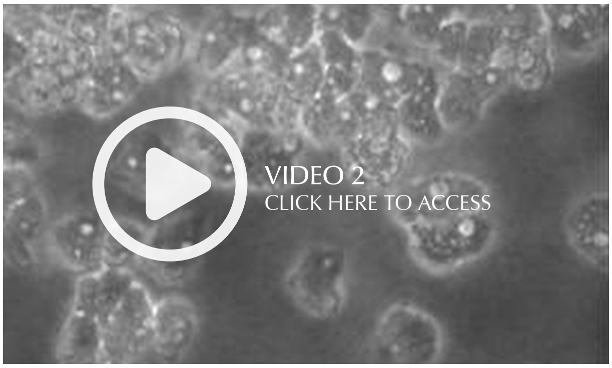

Video 2: Uninfected *Acanthamoeba castellanii* cells as control for mimivirus infections.
